# Revisiting Thyroid Hormones in Schizophrenia

**DOI:** 10.1155/2012/569147

**Published:** 2012-03-26

**Authors:** Nadine Correia Santos, Patrício Costa, Dina Ruano, António Macedo, Maria João Soares, José Valente, Ana Telma Pereira, Maria Helena Azevedo, Joana Almeida Palha

**Affiliations:** ^1^Life and Health Sciences Research Institute (ICVS), School of Health Sciences, University of Minho, Campus de Gualtar, 4710-057 Braga, Portugal; ^2^ICVS-3Bs PT Government Associate Laboratory, Braga, Guimarães, Portugal; ^3^Department of Pathology, Leiden University Medical Center, Leiden, The Netherlands; ^4^Institute of Medical Psychology, Faculty of Medicine, University of Coimbra, Coimbra, Portugal

## Abstract

Thyroid hormones are crucial during development and in the adult brain. Of interest, fluctuations in the levels of thyroid hormones at various times during development and throughout life can impact on psychiatric disease manifestation and response to treatment. Here we review research on thyroid function assessment in schizophrenia, relating interrelations between the pituitary-thyroid axis and major neurosignaling systems involved in schizophrenia's pathophysiology. These include the serotonergic, dopaminergic, glutamatergic, and GABAergic networks, as well as myelination and inflammatory processes. The available evidence supports that thyroid hormones deregulation is a common feature in schizophrenia and that the implications of thyroid hormones homeostasis in the fine-tuning of crucial brain networks warrants further research.

## 1. Introduction

In 1888 a report by the Committee of the Clinical Society of London explored the association of hypothyroidism with psychosis [1]. Not surprisingly, given the essential role of thyroid hormones for mammalian brain development, the effect of thyroid hormones (THs) in the modulation of affective illness and behavior continues to be an avenue of research in the pathophysiology of mood disorders [2–12]. Complementarily, research has revealed the TH modulation of crucial brain neurotransmitter systems [12–15] including the dopaminergic, serotonergic, glutamatergic, and GABAergic networks [14, 16–20]. As elaborated on throughout this paper, the misregulation of these pathways, as well as the participation of myelination and of cytokines, is of particular relevance in the schizophrenic brain [18, 21–23]. 

Schizophrenia is one of the most severe psychiatric disorders with an estimated prevalence of 0.7–1.0% in the population worldwide. It often runs a chronic and debilitating course, with many patients responding poorly to medication and suffering frequent and disrupting relapses. Furthermore, it is accompanied by a great social cost in terms of productivity loss and treatment-related expenses [21]. Its core features include cognitive impairment, delusions, hallucinations, altered volition and emotional reactivity and disorganized behavior. It is now clear that the heterogeneity and complexity of schizophrenia is both at the clinical and aetiological levels and that this complex disorder arises from the interaction of a range of deviant genetic traits and environmental “insults,” which may begin to act in the prenatal period [21]. The clear understanding of schizophrenia's molecular mechanism(s) is elusive, and no biological marker has been identified. In effect, a biomarker may be difficult to find if the disease results from a subtle deregulation in a biological network with impact on mental health and behavior. In this context, modulators of transcriptional activity and their carriers/receptors are good candidates in bridging the genetic and environmental determinants of schizophrenia. Among these are TH [24].

Circulating THs, the prohormone thyroxine (T_4_) and the active metabolite 3,5,3′-triiodothyronine (T_3_), are lipophilic molecules carried by plasma or cerebrospinal fluid (CSF) proteins. These molecules exert their function mostly via thyroid receptors that bind T_3_ with high affinity, acting as ligand-inducible transcription factors that regulate expression of T_3_-responsive genes. Nonetheless, TH may also act through fast nongenomic actions [25]. The timing and adequate amount of TH's action is crucial for the normal neurodevelopment and maturation of the central nervous system (CNS) and for proper functioning of the adult brain [6]. Given their described roles, it is not entirely unexpected that a link between TH and psychiatric disease may be considered [24, 26]. In the adult, TH fluctuations are associated with mood alterations, such that changes in TH levels are common in psychiatric patients across all ages [27–34]. Clinical case reports reveal that hyperthyroid individuals may manifest psychosis and depression [34–38]. Additionally, the prevalence of clinical hypothyroidism in psychiatric patients ranges from 0.5% to 8% [34, 38]. In fact, hypothyroid patients, or those with hypothyroxinemia, display mood impairment, as well as decreased motivation and increased depressive symptoms, such that the prevalence of depressive symptoms is close to 50% in people with hypothyroidism [34, 38]. In this regard, studies have evidenced the impact (correlation) of TH fluctuations in depression, including the relation between TH levels and depressive-state and treatment outcome and/or response time to treatment [39–50]. “Of notice, TH seem important for the mood improvement” ability of antidepressants, since about 50% of treatment-resistant patients become responsive when TH are coadministered. In particular, T_3_ administration has been recognized to hasten recovery [51, 52], although adjunctive supraphysiological doses of T_4_ have proven especially efficacious in women with unipolar or bipolar disease and refractory to standard medication [13]. In addition, dementia has been reported in cases of severe hypothyroidism [34, 38].

In this paper, data on TH circulating levels in cohorts of schizophrenic patients will be summarized. Also, we will explore the interplay between TH and main biological networks implicated in schizophrenia.

## 2. Thyroid Hormones and Schizophrenia: Relationship with Neurotransmitter Systems and Neural Networks

The link between TH and schizophrenia is pertinent [24, 26, 53]. In point of fact, several groups have measured TH levels, and other thyroid-related parameters, in schizophrenic patients (hospitalized and outpatients), reporting on several abnormalities. A compilation of these studies is presented in Table 1. For the collection of the presented reports the PubMed database was searched using key terms such as “psychiatric disease,” “schizophrenia,” and “thyroid hormones [levels],” and/or designation of each thyroid hormone specifically [total and free thyroxine (TT_4_ and FT_4_) and triiodothyronine (TT_3_ and FT_3_), and thyroid stimulating hormone (TSH)]. Subsequently, for the generation of the final list, the PubMed-generated list was cross-referenced with that derived from the bibliography in articles on schizophrenia and/or psychiatric disease and TH. From our analysis, to date, 15 independent studies of human population cohorts have been published addressing the role of TH in schizophrenic patients, with assessments and/or measurement of TH status [28, 33, 54–66]. It is of mention that prior to the mid-1980s the lack of high-sensitivity assays for measurement of TH, specifically for free TH, was a handicap. Nonetheless, earlier reports already made mention of thyroid function abnormalities in schizophrenic patients and their families, as well as on the resemblance between the psychotic symptoms of people with severe hypo- and hyperthyroidism and those of schizophrenic patients [67–69].

Altogether, from the literature analysis, a dynamic relationship emerged. For example, a connection between Brief Psychiatric Rating Scale (BPRS) values, TT_4_ and FT_4_ levels and clinical recovery in male psychiatric patients was reported [58]. Also, a study by Roca and colleagues [60] evidenced that 49% of psychiatric patients, in their study population, exhibited significant changes in one or more TH levels, with a significant positive correlation between severity of illness and elevations of TH levels. Furthermore, clinical case reports indicate that hyperthyroid individuals may manifest psychosis [35–37], a characteristic of the positive symptoms observed in schizophrenic patients [21], and that hypothyroid individuals display mood impairment, such as decreased motivation and increased depressive symptoms, a presentation similar to the negative symptoms in schizophrenic individuals [21]. Adding to these, unpublished work from our laboratory indicates that, even within normal range, FT_3_ levels in Portuguese schizophrenic male and female patients were significantly lower when compared to controls and that FT_4_ levels were significantly lower in male patients when compared to mentally healthy individuals (data presented in meeting proceedings [70]).

Despite difficult interpretation, methodological limitations, and heterogeneity of patients, including complex history of antipsychotic medication, overall observations indicate that TH level fluctuations may have clinical meaning. Such observations are pertinent given that the interaction between the pituitary-thyroid axis and the dopaminergic, serotonergic, glutamatergic, and GABAergic systems, together with any correlation with myelination and proinflammatory response, are relevant in schizophrenic patients in light of their implications in the etiology of the disease [14, 16–20, 22, 23, 71]. In the schizophrenia context, each of these neural networks will be next addressed.

### 2.1. Dopaminergic System

Dopamine was the first neurochemical to be associated with schizophrenia, in part due to the efficacy of antipsychotic drugs that block dopamine D2-like receptor in alleviating the hallucinations and delusions of patients (review [19]). Additionally, neuroimaging studies have revealed enhanced activity of the nigrostriatal dopamine system, albeit a hypofunctionality of the mesoprefrontal cortical system, in schizophrenic individuals [72, 73].

Thyroid hormones have been shown to regulate the levels of dopamine receptors [74, 75] and the activity of tyrosine hydroxylase [76–78], the rate-limiting enzyme of the cathecolaminergic pathway. Moreover, it has been suggested that dopamine may be inhibitory of TSH secretion [59], as treatment with dopamine blockers lead to increase in TSH level or to subclinical hypothyroidism [79], and that hypothyroidism can lead to increased dopamine receptor sensitivity [74]. In a human study of acutely ill schizophrenic patients, Rao et al. [55] analyzed the interrelation between serum levels of dopamine, prolactin, TSH, and T_4_. The serum levels of dopamine were found to be elevated in schizophrenic patients, while levels of the other parameters were decreased. The increased dopaminergic activity was hypothesized to affect the pituitary secretory function, and decreased beta-adrenergic activity was inferred as consequence of decreased serum TSH concentration. This is of further interest as *α*1- and *β*-adrenergic catecholamines are involved in maintaining deiodinase activity, and thus brain thyroid status [80]. As such, type-1 deiodinase impairment may result in a drop in T_3_ levels, with unchanged T_4_, and type 2 or 3 deiodinase impairment may be reflected in decreased T_4_ metabolization.

### 2.2. Serotonergic System

Serotonin (5-hydroxytryptamine, 5-HT) is an essential neurotransmitter. Curiously, it was first thought to have a role in schizophrenia given its similarity to lysergic acid diethylamide (LSD), a compound that competes for and occupies serotonin's receptor sites, resulting in psychotic symptoms [81]. Since then, perhaps the strongest evidence of serotonin's involvement in schizophrenia is the role that its receptors play in the mechanism of atypical antipsychotic drugs which, besides their high receptor selectivity, show a weak direct dopaminergic antagonist effect [82]. According to the current view, serotonergic signaling may have a modulatory influence on central dopamine transmission, which may significantly contribute to the therapeutic effects of atypical antipsychotics [83]. Altogether, observations led to a serotonin hypothesis of schizophrenia. Enhanced serotonergic signaling, especially via serotonin type 2A receptors, is thought to be involved in the pathology of schizophrenia specifically during the early phases of psychoses ([84] and review [20]). On the other hand, deficient central 5-HT functions may underlie some of the negative symptoms in schizophrenic patients [83, 85].

Establishing a link between the serotonergic system and TH modulation, Strawn et al. [86] measured CSF concentrations of 5-hydroxyindoleacetic acid (5-HIAA) and homovanillic acid (HVA), major metabolites of serotonin and dopamine, as well as plasma concentrations of various THs. The concentration of 5-HIAA was significantly and negatively correlated with plasma TSH and TT_3_, while that of HVA was significantly and negatively correlated with plasma TSH, TT_3_, and FT_3_. Such findings, indicative of monoamine-thyroid interactions, are significant as studies have shown diminished 5-HT activity in hypothyroid patients [87, 88] and a negative correlation between TSH and CSF concentrations of 5-HIAA in patients with unipolar depression [89]. Of note, in a separate population of patients diagnosed with major depressive disorder, no correlation was found between TSH and 5-HIAA [90]. Nonetheless, as reviewed by Bauer et al. [13], most human studies in hypothyroid patients evidence a reduced 5-HT responsiveness, that is, reversible with TH replacement therapy. Furthermore, studies in hypothyroid-state-induced animals showed that 5-HT turnover is increased in the brainstem and that its levels, as well as those of its precursors, are decreased in the cortex/whole brain [91–94]. Also, reports indicate an increase in cortical 5-HT concentrations and desensitization (no change in density) of autoinhibitory 5-HT_1A_ receptors in the raphe area, resulting in disinhibition of cortical and hippocampal 5-HT release, and in increased cortical 5-HT_2_ receptor sensitivity [95–97]. Altogether, there is evidence that thyroid status impacts the serotonin system in the adult brain and vice versa [13].

### 2.3. Glutamatergic System

The glutamatergic hypothesis of schizophrenia is based upon the observation that psychotomimetic agents, such as ketamine and phencyclidine, induce neurocognitive deficiencies and psychotic symptoms, similar to those of schizophrenia, through blockage of the neurotransmission at N-methyl-D-aspartate-(NMDA-) type glutamate receptors [98]. Given that the glutamate/NMDA receptors are ubiquitously distributed in the brain, glutamatergic models of schizophrenia predict widespread cortical dysfunction, in particular hypofunctionality of the forebrain glutamate system (review [16, 99]). Furthermore, supporting the model in which reduced NMDA receptor activity may result in schizophrenic-like behavior, animal data have shown that mice expressing only 5% of normal levels of the NMDAR1 receptor subunit display behavioral abnormalities similar to those observed in pharmacologically induced animal models of schizophrenia [100]. The phenotype can be ameliorated by treatment with antipsychotic drugs (dopaminergic and serotonergic receptors antagonists). Altogether, the literature corroborates a link between the glutamatergic- and dopaminergic-based systems, particularly considering that the NMDA receptors are colocated on brain circuits that regulate dopamine release [99].

Mendes-de-Aguiar et al. [15] studied the role of T_3_ in the CNS, specifically on regulation of glutamate uptake. The team showed an increased neuronal viability against toxicity when neurons were cultured in the presence of T_3_-treated astrocytes. Altogether, the authors concluded that T_3_ is capable of regulating extracellular glutamate levels by modulating the astrocytic glutamate transporters and, consequently, by promoting neuronal development and neuroprotection. In another study, male rats were treated with glutamate receptor agonists and antagonists and serum TH levels were assessed [101]. The results indicated that agonist administration increased TSH concentrations, while antagonists decreased TSH and TH serum levels, indicating that endogenous excitatory amino acids may play a part in the regulation of TH secretion [102]. These studies are in agreement with reports on glutamate and other endogenous excitatory amino acids, such as L-aspartate, N-methyl-D-aspartate, kainate, and amino-hydroxy-5-methyl-4-isoxazole propionate, in their ability to regulate the secretion of anterior pituitary hormones as well as in the neuroendocrine regulation of the hypothalamic-pituitary axis (review [103]).

### 2.4. GABAergic System

The role for the GABA (*δ*-aminobutyric acid)-ergic system in the pathogenesis of schizophrenia derives mostly from neuropathologic studies [104]. Specifically, the chandelier neurons, a subtype of GABA interneurons, have decreased immunostaining for the GABA transporter, possibly related to decreased BDNF signaling or NMDA receptor hypofunction. Furthermore, upregulation of the postsynaptic GABA-A receptors, together with reduction of both glutamic acid decarboxylase (GAD) 67 and reelin (a protein that colocalizes with GABAergic interneurons), was described in schizophrenic patients [105]. GAD67 and reelin are involved in the glutamate conversion to GABA and in synaptic plasticity and/or neuromigration.

The possibility that TH affects the GABAergic system was first putforward in the 1960s and since then multiple studies have examined various aspects of this relationship, altogether suggesting that some human nervous disorders involving GABAergic systems are related to thyroid dysfunction. Overall, as expertly reviewed by Wiens and Trudeau [14], the effect of TH on the GABAergic system can take place at multiple levels, including circuit formation, enzymes involved in synthesis and metabolism of GABA and glutamate, GABA release and reuptake, and GABA receptors. For example, in rat models, thyroidal status has been shown to influence the development of inhibitory cortical GABAergic circuits [106]. Also, neonatal hypothyroidism was evidenced to result in decreased GAD activity in various brain regions of the neonate brain [107, 108], although not in the adult brain. In addition, T_3_ administration was shown to accelerate the developmental increase in GAD activity in both *in vivo* and *in vitro* models [109, 110]. Furthermore, other studies revealed lower activity of two other enzymes in GABA metabolism, GABA aminotransferase and succinic semialdehyde dehydrogenase, in hypothyroid animals [111], and that T_3_ replacement restored activity back to control levels [112]. At the GABA concentration level, neonatal rats rendered hypothyroid have reduced whole brain glutamate and GABA concentrations, from within 2 hours of birth to postnatal day 30 [113]; interestingly, levels were not found to be increased in animals rendered hyperthyroid [114]. In contrast, whole glutamate and GABA levels were found elevated in hypothyroid animals [115] (rendered hypothyroid when adults) and, in accordance, with the increase GAD activity also noted. These observations point for the possible diverse influence of TH depending on the developmental stage. Data for animals rendered hyperthyroid are discordant between studies. Overall, evidence continues to indicate a correlation, although a positive correlation between TH levels and GABAergic function in the developing brain and a negative correlation in adult animals is not always a consistent finding [14]. Adding to these observations, studies indicate that TH affects GABA release and reuptake. For example, in *in vitro* preparations of synaptosomes, from adult rat cerebral cortex, low concentration of T_3_, but not of T_4_ or rT_3_, increased depolarization-induced GABA release by a direct nongenomic mechanism [116]. At the GABA receptor level, studies indicate that TH have direct nongenomic effects on the GABA_A_ receptor complex, specifically that, in the presence of GABA, T_3_ inhibits GABA-stimulated Cl^−^ currents in rat forebrain membranes and in its absence it induces the Cl^−^ current [117, 118]. On the other hand, GABA can affect TH function; GABA can inhibit TSH-stimulated TH release from the thyroid gland and affect TSH secretion from the pituitary.

On the role of adequate functioning of the maternal thyroid gland in offspring thyroid status development, the report by Ahmed et al. [119], on hypo-/hyperthyroidism animal models in dams, is especially noteworthy regarding the TH-GABAergic system interplay. The study revealed that maternal hypothyroidism induced decreases in both monoamine levels and in acetylcholinesterase activity and increases in the GABA content of the offspring. This was accompanied by suppression of Na^+^, K^+^-ATPase, Ca^2+^-ATPase, and Mg^2+^-ATPase activity in different brain regions. On the other hand, maternal hyperthyroidism produced reverse effects. The authors concluded that maternal hypothyroidism and hyperthyroidism might induce inhibitory and stimulatory effects, respectively, on the excitability and synaptic neurotransmissions in the progeny's brain [119].

### 2.5. Myelination and Cytokines

The TH involvement in the regulation of myelination and/or oligodendrocytes' functionality, central processes in the modulation of neural networks, is of interest in schizophrenia, where involvement of white matter has been implicated [22, 71, 120–123].

Associations between TH levels and myelination have been reported. Hypothyroidism is associated with delayed myelination in several brain regions [124, 125]. Furthermore, myelin-related genes, shown to be downregulated in postmortem schizophrenic brain, including cyclic nucleotide phosphodiesterase, myelin-associated glycoprotein, transferring, and v-erb-b2 erythroblastic leukemia viral oncogene homolog 3 [122], are regulated by TH. Also, changes observed in the identified cell cycle genes, from microarray analysis of schizophrenic patients [123], are particularly interesting given that two genes, cyclin D1 and cyclin-dependent kinase inhibitor 1C (P57), central to oligodendrocyte differentiation, have been shown to be among the early regulated cell cycle genes after exposure to TH, a “cue” essential to trigger oligodendrocyte differentiation [126, 127].

Myelin abnormalities in the neurological/psychiatric-diseased brain are often presented with an inflammatory component. In schizophrenia, evidence from clinical data supports a potential pathogenic role of elevated cytokine expression. Both childhood and adult schizophrenia are characterized by elevated expression of IL-1, IL-6, and TNF-*α* in the CSF, along with altered cytokine or cytokine receptor expression [23, 128]. While many cytokines may be virtually undetectable in a healthy noninflamed system, their induction (abnormal or in a response to an inflammatory trigger) in immune and glial cells, such as astrocytes and microglia, may play a significant role in the deregulation of neural cell homeostasis, with vast consequences at the level of oligodendrocyte function and myelination (review [71]). In this line, it is relevant to mention that whereas THs play an important role in the regulation of deiodinases activity under normal metabolic conditions, other regulating mechanisms might be involved in TH metabolism during pathophysiological conditions, which may overlap with those known to be relevant for the development of schizophrenia. During these conditions, a state of altered TH metabolism can occur [nonthyroidal illness (NTI)], which is characterized by a fall of serum T_3_ [129, 130], due to decreased extrathyroidal conversion of T_4_ into T_3_ by type 1 deiodinase, without an increase in serum TSH [131, 132]. In these studies, TR*β*1 has been found to be downregulated (in an animal model of NTI), and type 3 deiodinase activity shown to be upregulated in liver and skeletal muscle of critically ill patients. Correspondingly, what renders these observations of particular interest, in the schizophrenia-TH-inflammation interrelation, is that in sites of local inflammation, induced in animal models by site-directed bacterial endotoxin (lipopolysaccharide) administration, deiodinase type 3 activity in inflammatory cells is strongly induced, suggesting enhanced local degradation of T_3_ [132].

### 2.6. Thyroid Hormones as Neurotransmitters

The role of TH in the pathophysiology of schizophrenia is more so noteworthy when considering the possible function of TH as neurotransmitters. The breakthrough hypothesis of a neurotransmitter role for T_3_ was put forward in the endocrinology field in the 1970s by Dratman and collaborators [133], based on the colocalization of TH with the noradrenergic system [134]. Given the vast roles of T_3_ in the brain, this is hardly unexpected. Among others, T_3_ promotes differentiation in astrocytes, mediates cerebellar astrocyte and neuronal proliferation, and participates in the organization of extracellular matrix molecules via astrocytes [15, 135]. Recently, Scanlan and team (review [136]) have explored a similar neurotransmitter function for 3-iodothyronamine (T(1)AM), a molecule proposed to result from a unique biosynthetic deiodination pathway starting from the decarboxylation products of either T_4_ or rT_3_. The hormone T_3_ is reported to accumulate in nerve endings reaching high concentrations in the synaptosome [137, 138] and being released from it in a Ca^2+^-dependent mechanism [139]. In *in vitro* studies T1AM has been found to block the transporters for the amines/neurotransmitters dopamine, norepinephrine, and serotonin. Interestingly, T1AM binds with high affinity to the trace-amine-associated receptor (TAAR) [136, 140], a class of G-protein-coupled receptors, and genetic linkage studies have shown a significant association between the TAAR gene and susceptibility to schizophrenia [141].

## 3. Thyroid Hormones and Schizophrenia: Human Studies Considerations

Thyroid hormones are widely distributed in the brain, with a multitude of effects on the CNS including a putative effect in the pathogenesis of psychiatric disorders. Indicating this interrelation, the successful treatment of affective disorders often includes the coadministration of TH. Despite these observations, the molecular action(s) that may underlie the mood-modulating properties of TH in the adult brain has only fairly recently become of greater interest. As such, when reporting on this type of analysis, three main aspects are of essence for the neuropsychiatric community to incorporate and/or consider in current and future studies: (i) the effect of antipsychotic medication, (ii) determination of CSF levels of TH, and (iii) the introduction of longitudinal studies of prenatal, neonatal, and/or childhood TH status, related to propensity to develop schizophrenia at the adult age, particularly in at-risk offspring (e.g., familial history of schizophrenia), as well as familial TH level correlations. These will next be summarily discussed.

### 3.1. Effect of Antipsychotic Medication on Thyroid Hormone Status

The literature reports on the effect of neuroleptic medication on deiodinases activities, as well as on the N-glucuronidation of TH, and by consequence on TH levels. Namely, the commonly used antipsychotic haloperidol can enhance type 2 deiodinase, while clozapine decreases type 2 but increases type 3 deiodinase activity in several brain regions [142]. In addition, some antipsychotics, such as clozapine, are piperazine-containing drugs that undergo N-glucuronidation. Given that the enzyme UDP-glucuronosyltransferase is responsible for the glucuronidation of TH and of certain psychotropic medications [143], a competitive mechanism may be conducive to TH level changes [65]. Finally, it may also be worthy of consideration that, even in cases where no change in circulating levels of TH is observed, deregulated deiodinase activity may affect the spatiotemporal distribution and local regulation of TH [144, 145].

### 3.2. Serum and CSF Thyroid Hormone Level Assessments

The measurement of TH levels in CSF samples would be more likely to represent TH brain homeostasis. Not only would this type of analysis add to ones already done to identify schizophrenia disease markers (e.g., [146] and review [147]), but it would also fill in a gap regarding the measurement of TH levels in the CSF of schizophrenic patients as such study is lacking in the field. This could be done in a manner similar to a study in an Alzheimer's disease population, which revealed rT_3_ level alterations in the CSF that were not reflected in the sera samples [148].

### 3.3. Familial, PreNatal, Neonate, and Early Childhood Thyroid Status

During development TH play a crucial role in CNS development, including in cerebral cytoarchitecture, neural growth, and synaptogenesis [149–151]. Consequently, it follows that the thyroid status and timing during development, including neonatal, has a significant impact on behavior, locomotor ability, speech, and cognition [2–8, 152]. Furthermore, given that the fetus relies on the mother for the adequate supply for TH, it is relevant to consider maternal thyroid status during pregnancy; for example, maternal hypothyroxinemia leads to decreased T_4_ availability for the fetal brain, that is, associated with neuropsychological impairments of the child [153]. Neurodevelopment can be restored to within the normal range upon TH supplementation in case of neonatal hypothyroidism [154], although subtle abnormalities remain in these children. Such observations are further correlated with animal work findings that indicate how TH status, even prenatally, may impact on neuronal excitability and synaptic transmission within the CNS [119]. Altogether, TH status seems relevant in the pre- and postnatal periods, and there are critical periods during which different parts of the brain and/or aspects of the CNS development are sensitive to TH supply [155]. Thus, to further understand the impact of TH on behavior, it would be of interest not only to investigate TH status in adulthood in mood and cognitive disorders, but also how alterations in the hormonal milieu during development might impact on adult behavior (as has been recently shown to be case for other hormones, such as glucocorticoids [156, 157]). In this regard, it would be further interesting to broaden thyroid assessment studies to include mentally healthy siblings of schizophrenia patients.

## 4. Conclusions

Thyroid hormone assessment in schizophrenic patients presents a particular challenge. Often, the heterogeneity of patients, including many with a complex history of antipsychotic medication, renders impossible a “clean” TH basal determination in disease state. Deregulations of the pituitary-TH axis continue to be of interest given the interaction between the pituitary-thyroid axis and the dopaminergic, serotonergic, glutamatergic, and GABAergic systems, together with relationships with myelination and proinflammatory response, which are strongly implicated in schizophrenia. The fine-tuning of these networks and their precise implication on disease etiology certainly warrants further investigation.

## Figures and Tables

**Table 1:** Literature review of studies on thyroid hormone levels in schizophrenic patients.

Serum analysis							Other parameters	
TT_4_	FT_4_	TT_3_	FT_3_	rT_3_	TSH	Antiperoxidase, Tab, or TBG	Number of patients and controls	Diagnosis criteria
No significant difference between controls and drug-free. Decreased after chlorpromazine, trifluoperazine, or clozapine treatments	No significant difference between controls and drug-free. Decreased after chlorpromazine and clozapine treatments						41 drug-free (for at least 3 weeks) SZ female (28) and SZ male (13) patients; 24/41 treated (6 weeks), random attribution, with chlorpromazine (10), trifluoperazine (9), or clozapine (5)	Feighner criteria 1972 and BPRS
*Notes and Conclusions.* FT4I calculated from multiplication of serum T_4_ and radioactive T_3_ uptake. Calls for investigation of serum TH levels before and after neuroleptic treatments (Rinieris et al., [54]).								

					Decreased		10 acutely-ill SZ patients, 10 healthy controls	
*Notes and Conclusions.* Levels of prolactin, and L-thyroxine decreased in SZ patients, whereas dopamine was elevated. Authors conclude that increased dopaminergic activity affects pituitary secretory function, and may result in decreased TSH (Rao et al., [55]).								

Basal within normal, posttreatment normal	Basal within normal, posttreatment normal				At 14 h, but not at 24 h, higher after treatment but within normal range		25 males drug-free SZ patients; treated (6w) with chlorpromazine (14) or fluspirilene (11)	Gorham criteria 1962 and DSM-III
*Notes and Conclusions.* Prolactin values higher (above normal range) posttreatment. Diagnosis of subclinical hypothyroidism in neuroleptic-treated SZ patients. Higher TSH basal level, and augmented TSH response to TRH, compared to pretreatment (Martinos et al., [56]).								

75% paranoid-SZ increased when compared with total 4% change (decrease) in the other groups	50% paranoid-SZ increased when compared with total 14% change (decrease) in other groups						29 males: 8 paranoid-SZ, 6 undifferentiated-SZ, 7 bipolar, and 8 major depression (chronic or subchronic patients)	SADS interview
*Notes and Conclusions.* Hospitalized patients. Measurement at admission and every 2 weeks thereafter (average 4 samples/patient). Analysis of variation within groups. Calls for longitudinal assessments of thyroxine trends even when within normal range levels (Mason et al., [57]).								

Overall falling levels during recovery. A subgroup with initial low levels that increased to middle-range values.	Falling levels during recovery. A subgroup with initial low levels that increased to middle-range values.						80 males: 6 undifferentiated-SZ, 9 paranoid-SZ, 18 schizoaffective, 15 bipolar disorder, 16 major depressive, 9 posttraumatic stress disorder, 7 others (chronic or subchronic)	BPRS and DSM III
*Notes and Conclusions.* Measurement of TT_4_ and FT_4_ at admission and at 2-week intervals. Indication that the existence of a change in TT_4_ and FT_4_, irrespectively of the direction, seems to be the most important parameter in recovery. No difference between diagnostic groups or treatments (Southwick et al., [58]).								

No significant difference between drugnaïve, drug-withdrawn, and controls		No significant difference between drug-naïve and drug-withdrawn; drug-naïve less than controls			No significant difference between drug-naïve, drug-withdrawn, and controls		23 drug-naïve SZ patients, 67 SZ with 2-3 days drug withdrawn, 67 SZ with neuroleptics; 90 controls	Schneider criteria 1987
*Notes and Conclusions.* Drug-naïve and drug-withdrawn groups combined due to similar hormone levels. Smokers removed only from controls. Dopamine increased in drug-free SZ patients compared to controls. Norepinephrine, epinephrine, and prolactin higher in neuroleptic-treated, compared to drug-free and controls. Agrees with hypothesis of dopaminergic overactivity in schizophrenia (Rao et al., [59]).								

Increased on day of hospitalization	Increased on day of hospitalization	Increased on day of hospitalization	Increased on day of hospitalization (negative correlation with BPRS)		Normal	TBG: Normal	15 male SZ and 34 female SZ patients; age-matched controls, 19 males and 34 females	DMS-III, BPRS and Montgomery-Asberg Depression scale
*Notes and Conclusions.* Measurements on day of hospitalization and after. Levels decreased later. Suggests that increases are due to increased T_4_ secretion by the thyroid (Roca et al., [60]).								

							26-year old SZ male, hospitalized 5 times with normal thyroid function and at a 6th with severe hypothyroidism. Haloperidol treatment.	
*Notes and Conclusions.* Thyroid function normalized with increased doses of medication. Managed hypothyroidism leads to decreased hospitalization. Hypothyroidism as a concurrent illness in SZ (Walch et al., [61]).								

Significantly lower in those with low or high TSH	FT_4_I significantly lower in those with low or high TSH	Low in 23% of patients with normal TSH values	FT_3_I significantly lower in those with low or high TSH		60% normal, 5% elevated, 17% low	TAb: 20% of total patients (28% SZ female to 13% control; 14% SZ-male to 7% control)	249 patients with chronic SZ (136 males, 113 females; median age 36 years old)	
*Notes and Conclusions.* Spectrum of thyroid function test abnormalities, but interpretation not clear (Othman et al., [33]).								

Normal		Mesor (daily mean): drug free lower than controls			Mesor (daily mean): SZ drug-free equal to treated and both lower than controls. Acrophase (daily higher), less than half in both SZ groups compared to control		89 drug-free SZ patients (21 never received drugs, remaining over 3days free), and 25 typical neuroleptic-treated SZ (for at least 5 days); 34 controls	Schneider criteria 1987 and Huber criteria 1987
*Notes and Conclusions.* Three independent psychiatrists. No age difference in TH measurements. TSH decrease might be caused by the hyperdopaminergic state of these patients as the group reported before. None on Li+; no more information on medication. Suggests involvement of the noradrenergic receptor system (Rao [62]).								

Increased in the acute SZ, normalized with perazine with 4-week treatment. Normal in all other SZ patients.		Normal		Normal	Normal		31 acutely ill SZ patients, 19 in remission without drugs, 20 in remission with different drugs, 24 with residual-SZ (negative symptoms); 24 controls	DMS-III revised and BPRS
*Notes and Conclusions.* Does not differ female from male. The higher T_4_ the higher the severity of illness, and the better the response to treatment. Concludes that function of T_3_ is altered as a consequence of neuroleptic therapies (Baumgartner et al., [63]).								

	Higher in patients with mild or major syndrome		Higher in patients with mild or major syndrome					BPRS
*Notes and Conclusions.* No clinical thyroid illness. 34% of patients with thyroid function tests abnormalities, and no correlation found with neuroleptic use. Calls for careful interpretation of thyroid function tests in SZ patients (Sim et al., [28]).								

		Higher in RP compared to controls	Higher in RP compared to controls		Higher in NRP compared to RP. Blunted response rate (TRH test) in RP group higher than NRP and control groups.		58 SZ patients, divided into a “remitted (RP)” (30) and “nonremitted (NRP)” (28) groups; 30 healthy controls	Mental deterioration battery (at regular intervals for 1 year)
*Notes and Conclusions.* Basal prolactin higher in RP, but not NRP, compared to controls. Basal growth hormone (GH) higher in NRP than RP SZ patients. Dexamethosone Suppression Test (DST) nonsuppression higher in RP than NRP and controls. Indicates that higher basal TSH and GH levels may be related with poorer treatment response. Higher TT_3_ and FT_3_, and blunted TSH response to TRH and nonsuppression in DST indicate better response in SZ patients (Yazici et al., [64]).								

2/22 (9%) baseline abnormal vales			4/22 (18%) baseline abnormal values		4/30 (13%) baseline abnormal values		38 treatment-resistant SZ patients receiving 6-weeks quetiapine, risperid,one or fluphenazine treatment	DSM-IV and BPRS
*Notes and Conclusions.* Little change in thyroid function except for a significant decrease in TT_4_ in quetiapine treatment possibly due to UDP-glucuronosyltransferase competition with the drug. No hypothyroidism noted. At baseline no TH differences between treatment groups. No control group. No monitoring of TH function in patients receiving quetiapine recommended. Probably no effect of risperidone or fluphenazine on TH (Kelly and Conley [65]).								

Normal	Normal	Normal		Normal	Normal	Antiperoxidase: normal	1077 individuals, 60–90 years old, enrolled in a MRI study. No dementia in the beginning. Serum collected at enrollment. 6-year followup for dementia and MRI scan. No further TH status assessment.	Minimental, Geriatric Mental Scale Schedule and DMS-III
*Notes and Conclusions.* Even though atrophy is present in demented patients, the presence of atrophy does not necessarily lead to dementia. Lower TSH with anti-TPO associated with 4 times higher risk of dementia but *P* > 0.05. No association of serum TSH, FT_4_, TT_3_, rT_3_, anti-TPO with dementia. Higher FT_4_ and rT3 related with higher hippocampus and amygdala atrophy (de Jong et al., [66]).								

BPRS: Brief Psychiatry Rating Scale; DSM: Diagnostic and Statistical Manual of Mental Disorders; SADS: Schedule for Affective Disorders and Schizophrenia; TTR: transthyretin; TBG: thyroxine-binding globulin; TAb: thyroid antibodies; SZ: schizophrenia.
